# Retraction: Innovations and Challenges in the Development of COVID-19 Vaccines for a Safer Tomorrow

**DOI:** 10.7759/cureus.r204

**Published:** 2025-11-19

**Authors:** Devika S Kumar, Krishna Prasanth, Ashni Bhandari, Vivek Kumar Jha, Avula Naveen, Muthu Prasanna

**Affiliations:** 1 Research, Panimalar Medical College Hospital and Research Institute, Chennai, IND; 2 Department of Community Medicine, Sree Balaji Medical College and Hospital, Chennai, IND; 3 Department of Audiology and Speech Language Pathology, Shree Guru Gobind Singh Tricentenary (SGT) University, Haryana, IND; 4 Pharmacology and Therapeutics, All India Institute Of Medical Science Bilaspur, Bilaspur, IND; 5 Pharmaceutics, Pharmaceutical Biotechnology, Surya School of Pharmacy, Surya Group of Institutions, Villupuram, IND

The Editors-in-Chief have retracted this article. An investigation by the journal found evidence of authorship manipulation. The Editors-in-Chief therefore no longer have confidence in the provenance and reliability of the article contents. The authors disagree with the decision to retract.

